# Prosthetic joint infection diagnosis applying the three-level European Bone and Joint Infection Society (EBJIS) approach

**DOI:** 10.1007/s10096-022-04410-x

**Published:** 2022-03-22

**Authors:** Chiara Papalini, Giacomo Pucci, Giulia Cenci, Antonella Mencacci, Daniela Francisci, Auro Caraffa, Pierluigi Antinolfi, Maria Bruna Pasticci

**Affiliations:** 1 Infectious Diseases Clinic, Department of Medicine, University of Perugia, Santa Maria Della Misericordia Hospital, 06100 Perugia, Italy; 2 Internal Medicine, University of Perugia, Santa Maria Hospital, Terni, Italy; 3 Orthopedic Clinic, University of Perugia, Santa Maria Della Misericordia Hospital, Perugia, Italy; 4 Microbiology Laboratory, University of Perugia, Santa Maria Della Misericordia Hospital, Perugia, Italy; 5 Infectious Diseases Clinic, University of Perugia, Santa Maria Hospital, Terni, Italy

**Keywords:** Prosthetic joint infection, EBJIS, Diagnosis algorithm, Clinical, Microbiology

## Abstract

Sensitive and specific tests for the diagnosis of prosthetic joint infection (PJI) are lacking. The aim of this study was to report clinical and microbiological findings of consecutive patients diagnosed with PJI at the University Hospital of Perugia, Perugia, Italy, and to validate these diagnoses utilizing the European Bone and Joint Infection Society (EBJIS) three-level diagnostic approach from 2021. Patients with a PJI diagnosis were included in this study and examined retrospectively. Overall, 133 patients were diagnosed with PJI: mean age 72 years, 54.9% female, and 55.6% with more than one comorbidity. The most frequent involved joints were hip 47% and knee 42%. Aetiology was identified in 88/133 (66.2%): staphylococci resulted the most frequent microorganisms and over 80% (45/54) resulted rifampin susceptible. Applying the EBJIS approach, PJI diagnosis resulted: *confirmed* in 101 (75.9%), *likely* in 25 (18.8%), and *unlikely* in 7 (5.3%). *Likely* PJIs aetiology was *Staphylococcus aureus* 11/25, coagulase-negative staphylococci 8/25, *Streptococcus agalactiae* 3/25, viridans group streptococci 2/25, and *Pseudomonas aeruginosa* 1/25. No statistically significant differences were detected among the three diagnosis groups with regard to clinical characteristics with the exception of a higher number of *confirmed* PJIs occurring < 3 months after implantation. The logistic regression analysis did not disclose any independent predictor of *confirmed* PJIs. We recommend using all the diagnostic tests available to approach PJI diagnosis, and suggest caution before rejecting PJI diagnosis in the presence of highly virulent microorganisms from a single sample, in patients without sinus tract, and in those receiving antimicrobial at the time microbiologic samples are collected. Study approved by Umbrian Regional Ethical Committee, Perugia, Italy, Prot. N. 23,124/21/ON of 10.27.2021.

## Introduction

Joint replacement surgery is a widely performed procedure and it improves quality of life. However, up to 10% of recipients develops some complications over their lifetimes [1]. Prosthetic joint infection (PJI) is one of these complications, usually occurring in < 1–2% of primary arthroplasties [2–7]. PJIs are associated with a significant burden for both the patient and the community as they necessitate complex treatment and prolonged hospitalization [8, 9]. PJIs can also lead to unsatisfactory functional results or even permanent disability [2]. Additionally, there are important medico-legal implications for physicians.

Despite several guidelines developed by scientific societies [10–19], a PJI diagnosis is difficult to achieve, especially in low-grade infections, in immunocompromised patients, and/or patients with PJI but not undergoing surgery [19]. Errors in the diagnostic process may lead to unsatisfactory treatment results [20].

The objective of this study was to report on clinical and microbiological findings of PJIs diagnosed at the University Hospital of Perugia, Perugia, Italy, and to evaluate PJI diagnoses using the 2021 European Bone and Joint Infection Society (EBJIS) three-level approach [19].

## Materials and methods

### Study methodology

Retrospective review of PJI cases.

### Study population

Consecutive patients who were 18 years old or older, with PJI diagnosis between January 2005 and December 2019, were reviewed.

On admission, every patient signed an informed consent allowing for the use of de-identified collected data. Study design was in accordance with the Helsinki Declaration of the World Medical Association.

Diagnostic and treatment strategies were independent from the study.

PJIs were diagnosed on clinical, laboratory, and radiological findings by the caring physicians. No specific guidelines were followed. Microbiological workup was carried out according to our standardized hospital protocol.

The clinical charts of included patients were reviewed by two different study investigators (CP and GC). They registered demographics, clinical findings, and results of the following tests: erythrocyte sedimentation rate (ESR), C-reactive protein (C-RP), radiology, nuclear imaging and microbiology tests, type of surgery, and medical treatment. They confirmed PJI diagnosis and classified each case among one of the three different groups according to the 2021 EBJIS.

### Microbiological protocol

#### Intra-operative tissues, synovial fluid, and prosthesis

Intra-operative samples consisted deep tissues, joint fluid, bone fragments, and prosthesis. Tissue samples were taken variably in number, from 1 to 7, and inoculated into chocolate, blood, Sabouraud and Shedler agar plates, and BD BACTECTM Plus Aerobic/F and BD BACTECTM Lytic/10 Anaerobic/F bottle broth cultures (Becton Dickinson, Sparks, MD, USA). Aerobic and anaerobic cultures were incubated for 7 days. Synovial fluid aspirate was cultured using the same media and in selected cases, it was also examined for cells count. The prosthesis and any removed material were soaked with Muller-Hinton broth, vortexed for 5 min. Thereafter, 100 µl of broth were cultured into agar and broth media for 7 days. From 2011, the laboratory replaced vortexing with sonication [2, 21].

Sonication culture results were interpreted as follows: (1) ≥ 5 colony forming unit (CFU) confirmed criteria and (2) < 5 CFU or growth only broth cultures taken in consideration only for patients on antibiotic treatment at surgery, having previous similar antimicrobial investigations or Septi-Fast® results or the patients benefited from a treatment based on the microbiological results.

#### Blood cultures

In case of patients’ temperature ≥ 38.0 °C, blood cultures were collected using a BD BACTECTM Plus Aerobic/F and a BD BACTECTM Lytic/10 Anaerobic/F bottles (Becton Dickinson, Sparks, MD, USA). Positive cultures were processed for Gram staining and subcultures on solid media.

#### Swab cultures

In the presence of sinus tract, when swabs were collected, the drainage was cultured using chocolate, blood, and Sabouraud and Shedler agar plates for 48 h.

#### Molecular diagnostic methods

Up until 2019, in selected cases, either synovial fluid or sonication fluid were also examined using a real-time polymerase reaction with the Septi-Fast ® (SF) (Roche Diagnostic GmbH, Mannheim, Germany) [22, 23].

#### Bacterial identification

Colonies were identified following the procedures in use in the laboratory. From 2018, the MALDI Biotyper1 instrument (Bruker Daltonik GmbH, Bremen, Germany) has been used for bacterial identification. Antimicrobial susceptibility tests were performed with the BD Phoenix TM Automated Microbiology System (Becton Dickinson, Sparks, MD, USA) and interpreted according to current guidelines [24].

#### EBJIS classification

According to the 2021 EBJIS criteria, PJI can be classified as follows:

1. *Confirmed*, when at least one of the following findings is present: (i) cutaneous sinus tract communicating with the prosthesis or visualization of the prosthesis, (ii) increased leukocyte count and differential in the synovial fluid, (iii) alpha-defensin positive immunoassay or lateral flow assay, (iv) two positive samples with the same microorganism, (v) histopathological characteristics of acute inflammation with ≥ 5 neutrophils in ≥ 5 high power fields, and (vi) positive culture of peri-prosthetic tissue or sonication fluid;
2. *Likely*, when present at least one of the (i) radiological signs of loosening within the first 5 years after implantation, (ii) previous healing wound problems, (iii) history of recent fever or bacteraemia, (iv) purulence around the prosthesis, and (v) C-RP > 1 mg/dl, along with another finding of (i) increased leukocyte count and differential in the synovial fluid, (ii) positive culture of synovial fluid, (iii) single intra-operative culture, (iv) > 1 CFU/ml from sonication fluid, (v) histopathological characteristics of acute inflammation with ≥ 5 neutrophils in a single high power field observation, and (vi) positive WBC scintigraphy;
3. *Unlikely*, when (i) clinical features, (ii) synovial fluid cytological analysis, (iii) microbiology, (iv) histology, and (v) three-phase isotope bone scan imaging are negative [19].

### Statistical analysis

Continuous variables were expressed as mean ± standard deviation (SD) if normally distributed, or as median and interquartile range (IQR) if non-normally distributed. Categorical variables were expressed by their relative (%) frequencies. Difference between categories (*confirmed*, *likely*, or *unlikely* PJIs) was analysed through one-way ANOVA for continuously distributed variables and with chi-square test for categorical variables. A further post hoc test using the Bonferroni correction to evaluate the significance of head-to-head differences. A stepwise multivariate logistic regression model was built to analyse potential determinants of *confirmed* vs non-confirmed (*likely* + *unlikely*) PJI diagnoses, introduced in the model as dependent variable. In this model, joint involved, comorbidities, diabetes, interval between prosthesis implant and onset of symptoms (in days), onset of symptoms and diagnosis (in days), and aetiology were all included in the model as potential independent variables. Analyses were performed using SPSS software for Windows (version 22.0; IBM Corp., Armonk, NY), with significance set at a 2-sided *p* < 0.05.

## Results

Overall, 133 patients with PJI diagnosis were included in this study. The mean age was 72 years, 54.9% (73/133) were females, and 55.6% (74/133) had more than one comorbidity. The infected joints resulted: hip 47% (63/133), knee 42% (56/133), shoulder 9.8% (13/133), and ankle 0.7% (1/133) (Table 1).

**Table 1:** Patient characteristics

Patients *N* 133	
Mean age	72.1 years (SD 10.6)
Sex *N* (%)	M 60 (45.1)–F 73 (54.9)
≥ 2 Comorbidities *N* (%) 1 Comorbidity *N* (%) N. comorbidities *N* (%)	74 (55.6) 38 (28.6) 21 (15.8)
Type comorbidity *N* (%)	Diabetes 39 (29.3) Cardiovascular diseases 33 (24.8) Cancer 14 (10.5) Non end-stage renal failure 9 (6.7) Immunosuppressed^a^ 5 (3.7)
Type of joint *N* (%)	Hip 63 (47.4) Knee 56 (42.1) Shoulder 13 (9.8) Ankle 1 (0.7)
Classification EBJIS 2021 N (%)	*Confirmed* 101 (75.9) *Likely* 25 (18.8) *Unlikely* 7 (5.3)

Legend: ^a^One HIV-infected patient, 4 patients treated with > 10 mg/day of prednisone

According to the proposed EBJIS three-level approach, PJIs were classified as *confirmed* 101/133 (75.9%), *likely* 25/133 (18.8%), and *unlikely* 7/133 (5.3%) (Table 2). In regard to *confirmed* PJIs, clinical findings were diagnostic in 80%, and microbiology was performed in 76/101 (75%) with positive results in 62%. All 25 patients with *likely* PJI had at least one positive clinical finding and a single sample pre- or intra-operative positive culture. In the seven cases classified as *unlikely* PJI, pre- and/or intra-operative microbiologic investigations were negative. One of these patients had positive three-phase isotope bone scan; however, he was included in this group because he was lacking of any other criteria for *likely* or *confirmed* cases. These patients with *unlikely* PJIs were treated as follows: two 2-stage exchange, one DAIR, and four received medical therapy without surgery. At 1-year follow-up, 6/7 were free of symptoms, and 1/7 died for complications related to immunodeficiency. All were judged to be affected with PJI lacking alternative diagnosis for prosthesis malfunctioning and improving with specific treatment for PJI.

**Table 2:** EBJIS criteria applied to PJIs cohort

Test	*Confirmed* PJIs *N* 101 (%)		*Likely* PJIs *N* 25 (%)		*Unlikely* PJIs *N* 7 (%)	
	Performed	Positive	Performed	Positive	Performed	Positive
Clinical and blood workup	101 (100)	81 (80)	25 (100)	25 (100)	No alternative reason for implant dysfunction in all 7 (100)	
Synovial fluid cytological analysis	2 (2)	2 (2)	0 (0)	0 (0)	0 (0)	0 (0)
Synovial alpha-defensin	1 (1)	1 (1)				
Microbiology	76 (75)^a^	63 (62)^b^	25 (100)	25 (100)	7 (100)	0 (0)
Histology	1 (1)	1 (1)	0 (0)	0 (0)	0 (0)	0 (0)
Nuclear imaging			1 (4)	0 (0)	2 (28.6)	1 (14)^c^

Legend: ^a^25/101 inadequate samples, ^b^in 13/76, microorganism grew from broth and not from solid media, ^c^considered as *unlikely* PJI because lacking of any criteria belonging to the other 2 groups

*Staphylococcus aureus* (*S. aureus*) was identified in 27 cases, specifically: *confirmed* PJIs 16/27 and *likely* PJIs 11/27 (Table 3). Among the 11 *S. aureus* in *likely* PJIs, seven were isolated from the synovial fluid and in four from a percutaneous peri-prosthetic collection. Of the 11 *likely* PJIs, four received surgical drainage and prosthesis retention (DAIR), one 2-stage exchange, and 6 only medical treatments.

**Table 3:** Microorganisms

Microorganisms	*Confirmed* PJIs	*Likely* PJIs	*Unlikely* PJIs	Total
	*N*	*N*	*N*	*N* (%)
*Staphylococcus aureus*	16	11		27 (20.3)
Coagulase-negative staphylococci	19	8		27 (20.3)
*Streptococcus* spp.	5	5		10 (7.5)
*Enterococcus faecium*	1			1 (0.8)
*Cutibacterium acnes*	5			5 (3.7)
*Corynebacterium striatum*	4			4 (3)
*Bacillus licheniformis*	1			1 (0.8)
*Pseudomonas aeruginosa*	1	1		2 (1.5)
*Escherichia coli* ^a^	1			1 (0.8)
*Enterobacter cloacae* ^b^	1			1 (0.8)
*Acinetobacter baumannii*	1			1 (0.8)
*Candida* spp.^c^	2			2 (1.5)
*Mycobacterium abscessus*	1			1 (0.8)
Poly-microbial infection^d^	5			5 (3.7)
Uncertain aetiology^e^	36			36 (27)
Culture negative	2		7	9 (6.7)

Legend: ^a^ESBL producer, ^b^AmpC producer, ^c^one patient C. albicans and one patient C glabrata, ^d^Clostridium perfrigens + Staphylococcus capitis + Staphylococcus epidermidis, Enterococcus faecium + Acinetobacter baumannii + P. aeruginosa, Actinomyces neuii + Staphylococcus lugdunensis, Peptoniphilus harei + Staphylococcus epidermidis, and Candida albicans + Staphylococcus epidermidis, ^e^microorganisms grown from sinus swabs, broth culture, and single sample

Coagulase-negative staphylococci (CoNS) were identified in 27/133 (20.3%): 19 in *confirmed* PJIs and 8 in *likely* PJIs. Of the latter group, all the microorganisms were isolated from the synovial fluid culture. Two patients were treated with a 2-stage exchange approach, one with 1-stage exchange, one with DAIR, and four with antibiotic therapy alone.

In regard to staphylococci susceptibility, we observed the following rates: *S. aureus* 70.4% (19/27) oxacillin, 66.6% (18/27) levofloxacin, and 85.2% (23/27) rifampin; CoNS 25.9% (7/27) oxacillin, 33.3% (9/27) levofloxacin, and 81.5% (22/27) rifampin.

Streptococci were identified in 10 patients: 5 *confirmed* PJIs (3 *Streptococcus gallolyticus*, 1 *S. gordonae*, and 1 *S. mitis*) and 5 *likely* PJIs (3 *S. agalactiae*, 1 *S. gordonae*, and 1 *S. mitis*). *Likely* PJIs were treated with 2-stage exchange two cases, DAIR another two, and only medical therapy one.

*Pseudomonas aeruginosa* was identified in 2 patients: 1 with *confirmed* and 1 with *likely* PJI. This was an old patient treated for traumatic femoral fracture presenting with acute onset of fever and positive synovial fluid culture*.* The patient received drainage and antibiotic therapy.

Five patients (3.7%) had a poly-microbial infection caused by *Clostridium perfrigens* + *Staphylococcus capitis* + *Staphylococcus epidermidis*, *Enterococcus faecium* + *Acinetobacter baumannii* + *P. aeruginosa*, *Actinomyces neuii* + *Staphylococcus lugdunensis*, *Peptoniphilus harei* + *Staphylococcus epidermidis*, and *Candida albicans* + *Staphylococcus epidermidis.*

For 27% (36/133) of the cases, despite satisfying the criteria of *confirmed* PJIs, aetiology was uncertain. In these cases, microorganisms were identified from swabs in 22, broth cultures of sonication fluid in 7, and one single intra-operative sample in 7 cases. In 6/7, *S. aureus* grew from the unique intra-articular sample: 5 of these individuals had *S. aureus* positive blood cultures, too.

Finally, in two confirmed PJIs, cultures resulted negative.

We observed significant differences between groups in the time interval between implant and symptoms onset. Specifically, we found a significantly higher proportion of *confirmed* PJIs with symptoms onset < 3 months after implantation, as compared with *likely* and *unlikely* PJIs (Table 4). Also, considering the 54/133 staphylococcal infections, we noticed no statistical significance (*p* = 0.39) in rate of PJIs caused by *S. aureus* between *confirmed* (45.7%, 16/35) and *likely* PJIs (58%, 11/19).

**Table 4:** Clinical characteristics of the population according to EBJIS diagnostic criteria for PJIs*

Clinical features	*Confirmed* PJIs *N* 101	*Likely* PJIs *N* 25	*Unlikely* PJIs *N* 7	*p*
Time implant-symptoms	*N* (%)	*N* (%)	*N* (%)	
< 3 months 3–24 months > 24 months	59 (58.4) 18 (17.8) 24 (23.8)	10 (40) 10 (40) 5 (20)	1 (14.4) 3 (42.8) 3 (42.8)	0.04
Joint	*N* (%)	*N* (%)	*N* (%)	
Hip Knee Shoulder	53 (52.5) 36 (35.6) 11 (10.9)	9 (36) 15 (60) 1 (4)	1 (14.3) 5 (71.4) 1 (14.3)	0.195
Comorbidities	*N* (%)	*N* (%)	*N* (%)	
0 1 > 1	13 (12.9) 30 (29.7) 58 (57.4)	7 (28) 7 (28) 11 (44)	1 (14.3) 1 (14.3) 5 (71.4)	0.195
Diabetes	*N* (%)	*N* (%)	*N* (%)	
	31 (30.7)	6 (24)	2 (28.6)	0.818
Time symptoms-diagnosis (median in days)	30 (10–98)	30 (18–93)	75 (15–288)	0.231

^*^Continuous data are reported as median (interquartile range). *p* < 0.05 vs other groups

When the entire population was divided into those receiving versus not receiving a diagnosis of *confirmed* PJI, none of the examined variables was found to be an independent predictor of *confirmed* PJI diagnosis.

## Discussion

This study reports on clinical characteristics of a cohort of consecutive patients diagnosed with PJI from a single academic hospital and the results of PJI diagnoses utilizing the 2021 EBJIS three-level algorithm.

A timely and correct diagnosis of PJI is essential in order to achieve the best outcome. However, no single test has the absolute accuracy to diagnose it, and to identify the microorganism causing the infection.

In the cases of low-virulent microorganisms, too often, patients are seen after a long interval from the time of symptoms onset, the administration of an empirical antibiotic therapy, and with/without anti-inflammatory drugs. The delay leads to negative or inconclusive microbiologic investigations [20, 25, 26], adverse effects on biochemical serum markers [27], additional workup resulting in significant delayed diagnoses, and eventually more invasive surgeries.

We found that most of the PJI cases were diagnosed in poly-comorbid patients, with diabetes being the greatest risk factor. Hip and knee resulted the most involved joints and staphylococci were the most frequent identified microorganisms 54/133 (40.6%). These findings are in agreement with previous studies [28–30].

According to the 2021 proposed EBJIS approach, only 75.9% of our PJI diagnoses were *confirmed*; 18% (25/133) were classified as *likely* [19]. Of the 25 *likely* PJIs, 13/25 underwent surgery. However, microbiology at surgery was not done or results were not available; therefore, one criterium of *confirmed* PJI diagnosis was missed [19].

In our patients, antibiotic treatments were prescribed according to the in vitro susceptibility of the identified microorganisms when available.

Identification of the pathogens and their antibiotic susceptibility in vitro are one of the cornerstones for effective PJI treatment [10–19]. A correct intra-operative sampling is crucial to reach this aim. In 38 individuals of our cohort, PJI therapy was based on microbiological findings drawn from samples considered inadequate according to EBJIS criteria. In 6 of these cases, *S. aureus* was isolated, and 5/6 had also positive blood cultures. This kind of biological sample is not considered in EBJIS criteria. Anyway, in our opinion, these PJIs that we classified as of uncertain aetiology according to EBJIS guidelines should be considered PJIs caused by *S. aureus*.

The evidence of sinus tract is considered diagnostic of PJI; however, in most of the published literature, swabs from the sinus drainage are not accepted, due to non-concordance results between discharge and intra-operative or synovial fluid culture results in over 50% of the PJIs [31–34].

Molecular tests can be of further support for microorganism identification and are not affected by previous administration of antibiotics [2]. One of the limits of the molecular technique may be the lack of antimicrobial susceptibility as well as the lack of specificity, especially when CoNS are involved.

In literature CoNS are reported as the most frequent low-virulent pathogens causing chronic PJIs [25, 34–38]; nonetheless, they can also be potential contaminants [39]. Two positive peri-prosthetic cultures with the same microorganisms are considered confirmed criteria according to the majority of the available guidelines [17–20, 29, 30]. Likewise, at our centre, CoNS had to be identified in two different samples and had to have the same antibiotic susceptibility pattern to be considered true pathogens.

CoNS were also the most frequent microorganisms identified in poly-microbial PJIs.

Of note, in our cohort of patients, unusual pathogens, such as fungi [40] and non-tuberculous rapidly growing *Mycobacterium*, specifically *Mycobacterium abscessus* [41], were also identified which reinforce the importance of microbiologic investigation and microbiological diagnosis.

Regarding susceptibility results, almost 70% of *S. aureus* tested oxacillin susceptible and almost 80% rifampin susceptible, allowing for the administration of the most effective anti-biofilm treatment available. A lower rate of oxacillin susceptibility was observed among CoNS; in spite of this, over 80% resulted being rifampin susceptible.

Overall, PJI diagnosis based on 2021 EBJIS approach leads to a lower rate of PJIs diagnoses. Over 20% of our patients lacked criteria to confirm PJI diagnosis. The main reasons for this were (i) antimicrobial therapy not stopped before surgery, (ii) lack of intra-operative culture results, (iii) availability of a single microbiologic sample, (iv) lack of synovial fluid cytology, and (v) absence of histologic investigations.

Most of our *confirmed* PJIs were diagnosed within 3 months, being present a dehiscent wound and an exposed prosthesis, or after 24 months. These latter groups of patients presented with a sinus tract.

None of the clinical findings examined in our study was found to be a variable associated with a higher frequency of *confirmed* PJI diagnoses.

Being a retrospective study, including patients from a single centre, the 1-year follow-up limits the generalizability of our findings. In addition, investigations like alpha-defensin, synovial fluid cells count, and histology were not available or occasionally performed and several patients lacked intra-operative microbiology.

In conclusion, clinical-epidemiological data of our patients are in agreement with previous published studies. EBJIS definition of *confirmed* PJIs was applicable for 75% of our cases due to the fact that some of the useful investigations were not performed in our patients. We recommend using every available test approaching patients with suspected PJI and caution before rejecting PJI diagnosis in patients with acute PJIs caused by highly virulent microorganisms identified in a single sample, in patients presenting without sinus tract, and in patients receiving antimicrobial at the time microbiologic samples are collected.

## Data Availability

Anonymized datasets are available from the corresponding author on reasonable request.

## Abbreviations

CFU: Colony forming unit
CoNS: Coagulase-negative staphylococci
C-RP: C-reactive protein
DAIR: Debridement, antibiotics, irrigation, and retention of the prosthesis
EBJIS: European Bone and Joint Infection Society
ESR: Erythrocyte sedimentation rate
IQR: Interquartile range
PJI: Prosthetic joint infection
SD: Standard deviation
SF: Septi-Fast®
